# Draft genome sequence of *Vibrio parahaemolyticus* GISV1-1 associated with AHPND in Bangladesh

**DOI:** 10.1128/mra.00495-26

**Published:** 2026-08-13

**Authors:** Suzan Chandra Deb, Md. Rony Babu, Promi Saha, Umma Habiba Tanjum, Santu Biswas, Farzana Yeasmin, Arpita Guha, Md. Mustafizur Rahman, Md. Morshedur Rahman, Dipali Rani Gupta, Tofazzal Islam, Md. Mahbubur Rahman

**Affiliations:** 1 Institute of Biotechnology and Genetic Engineering, Gazipur Agricultural University 198780, Gazipur, Bangladesh; 2 Infectious Diseases Division, International Centre for Diarrhoeal Disease Research Bangladesh 56291, Dhaka, Bangladesh; 3 Department of Dairy and Poultry Science, Faculty of Veterinary Medicine and Animal Science, Gazipur Agricultural University 198780, Gazipur, Bangladesh; Indiana University Bloomington, Bloomington, Indiana, USA

**Keywords:** *Vibrio parahaemolyticus*, *Penaeus monodon*, AHPND, shrimp disease, virulence-associated gene

## Abstract

*Vibrio parahaemolyticus* GISV1-1 was isolated from diseased shrimp with acute hepatopancreatic necrosis disease in Bangladesh. Its draft genome is 5,078,728 bp with 45% GC content. The genome exhibits several virulence-associated genes and the beta-lactam resistance gene *blaCARB-33*. It will enhance our understanding of pathogenesis and disease management in shrimp aquaculture.

## ANNOUNCEMENT

The genus *Vibrio* comprises Gram-negative, halophilic bacteria that are widely distributed in marine and estuarine environments. Many species within this genus are recognized as pathogens of shrimp (1). Among them, *V. parahaemolyticus* is the primary etiological agent of acute hepatopancreatic necrosis disease (AHPND), which causes severe mortality in cultured shrimp (2). The emergence and global spread of AHPND-causing strains have resulted in significant economic losses to the shrimp farming industry worldwide (3).

*V. parahaemolyticus* strain GISV1-1 was isolated from the hepatopancreas of a naturally infected shrimp (*Penaeus monodon*) collected from a farm in Shamnagar, Satkhira (22.336661°N, 89.089998°E), Bangladesh, during an AHPND outbreak in June 2025. The diseased shrimp exhibited clinical signs consistent with AHPND, including lethargy, anorexia, reddish body, empty digestive tract, and a whitish hepatopancreas (4). Following shrimp surface disinfection with 70% ethanol, the hepatopancreas was aseptically removed, homogenized in sterile physiological saline, and serially diluted (10-fold). Thirty microliters of the 10^−4^ dilution were spread onto thiosulfate-citrate-bile salts-sucrose (TCBS) agar (Liofilchem S.r.l., Italy) and incubated at 25°C for 24  h. A representative green colony was streaked several times on nutrient agar (Liofilchem S.r.l., Italy) supplemented with 2% NaCl to obtain a pure isolate, designated GISV1-1.

Strain GISV1-1 was grown in 5 mL of nutrient broth overnight at 30°C with shaking at 200 rpm. Genomic DNA was extracted using the DNeasy Blood & Tissue Kit (Qiagen) following the manufacturer’s instructions. DNA quality and quantity were assessed via a NanoDrop (Thermo Fisher Scientific, USA), Qubit (Thermo Fisher Scientific, USA), and LabChip GX Bioanalyzer (PerkinElmer). Libraries were prepared with the Illumina DNA Prep Kit and sequenced on an Illumina NextSeq 2000 platform to generate 2 × 150 bp paired-end reads.

A total of 991,114 paired-end raw reads (approximately 59× coverage) were obtained. Read quality was assessed with FastQC v0.12.1 (5), and quality trimming was performed using Trimmomatic v0.39 (6) with the parameters LEADING:3 TRAILING:3 SLIDINGWINDOW:4:20 MINLEN:36. The draft genome was assembled using SPAdes v4.2.0 (7). Genome completeness was assessed via CheckM v1.2.4 (8), showing 97.56% completeness with 0.74% contamination. The assembly yielded 109 contigs, with a total length of 5,078,728 bp, a GC content of 45%, an N50 of 322,756 bp, and an L50 of 4. Default parameters were used for all software unless otherwise specified.

Annotation was performed using NCBI Prokaryotic Genome Annotation Pipeline (PGAP) v6.10 (9), which predicted 4,539 protein-coding genes, 66 tRNAs, 9 rRNAs, and 5 ncRNAs (Table 1). Virulence factor screening using Virulence Factor Database (VFDB) (10) identified several virulence genes including *VPA0450*, *ati2*, *acfB*, *lfhA*, *fliP*, *flhA*, *cheD*, *katA, cqsA,* and *tlh*. Resistance gene analysis using ResFinder 4.7.2 (11) detected beta-lactamase gene *blaCARB-33*, which is associated with resistance to beta-lactam antibiotics, including amoxicillin, ampicillin, and piperacillin. No acquired resistance genes were detected for other major antibiotic classes. PathogenFinder2 (12) predicted a high pathogenic potential (probability 0.8619) for humans.

**TABLE 1:** Genomic features of *Vibrio parahaemolyticus* strain GISV1-1

Genome features	Value
Genome size (bp)	5,078,728
GC content (%)	45
Number of contigs	109
N50 (bp)	322,756
L50	4
Total genes	4,654
Protein-coding genes	4,539
Total CDSs	4,574
Total RNA genes	80
tRNA genes	66
rRNA genes	9
ncRNA genes	5
Pseudogenes	35
Annotation pipeline	NCBI PGAP v6.10

The genome sequence represents an important genetic resource for elucidating virulence mechanisms and may facilitate the development of improved diagnostic tools and disease management strategies for shrimp aquaculture.

## Data Availability

The raw data are accessible under accession number JBSZAZ000000000. The data can be found under BioSample accession number SAMN53839609 and BioProject accession number PRJNA1378496. The raw data are available from the Sequence Read Archive under accession number SRR36540887.
